# Predicting Antituberculosis Drug–Induced Liver Injury Using an Interpretable Machine Learning Method: Model Development and Validation Study

**DOI:** 10.2196/29226

**Published:** 2021-07-20

**Authors:** Tao Zhong, Zian Zhuang, Xiaoli Dong, Ka Hing Wong, Wing Tak Wong, Jian Wang, Daihai He, Shengyuan Liu

**Affiliations:** 1 Department of Tuberculosis Control Shenzhen Nanshan Center for Chronic Disease Control Shenzhen China; 2 Department of Applied Mathematics The Hong Kong Polytechnic University Hong Kong China; 3 Department of Biostatistics University of California Los Angeles, CA United States; 4 Hong Kong Polytechnic University Shenzhen Research Institute Shenzhen China; 5 Research Institute for Future Food The Hong Kong Polytechnic University Hong Kong China; 6 Department of Applied Biology and Chemical Technology The Hong Kong Polytechnic University Hong Kong China

**Keywords:** accuracy, drug, drug-induced liver injury, high accuracy, injury, interpretability, interpretation, liver, machine learning, model, prediction, treatment, tuberculosis, XGBoost algorithm

## Abstract

**Background:**

Tuberculosis (TB) is a pandemic, being one of the top 10 causes of death and the main cause of death from a single source of infection. Drug-induced liver injury (DILI) is the most common and serious side effect during the treatment of TB.

**Objective:**

We aim to predict the status of liver injury in patients with TB at the clinical treatment stage.

**Methods:**

We designed an interpretable prediction model based on the XGBoost algorithm and identified the most robust and meaningful predictors of the risk of TB-DILI on the basis of clinical data extracted from the Hospital Information System of Shenzhen Nanshan Center for Chronic Disease Control from 2014 to 2019.

**Results:**

In total, 757 patients were included, and 287 (38%) had developed TB-DILI. Based on values of relative importance and area under the receiver operating characteristic curve, machine learning tools selected patients’ most recent alanine transaminase levels, average rate of change of patients’ last 2 measures of alanine transaminase levels, cumulative dose of pyrazinamide, and cumulative dose of ethambutol as the best predictors for assessing the risk of TB-DILI. In the validation data set, the model had a precision of 90%, recall of 74%, classification accuracy of 76%, and balanced error rate of 77% in predicting cases of TB-DILI. The area under the receiver operating characteristic curve score upon 10-fold cross-validation was 0.912 (95% CI 0.890-0.935). In addition, the model provided warnings of high risk for patients in advance of DILI onset for a median of 15 (IQR 7.3-27.5) days.

**Conclusions:**

Our model shows high accuracy and interpretability in predicting cases of TB-DILI, which can provide useful information to clinicians to adjust the medication regimen and avoid more serious liver injury in patients.

## Introduction

Tuberculosis (TB) is an infectious disease caused by the bacillus *Mycobacterium tuberculosis*. It is one of the top 10 causes of death worldwide and the leading cause of death from a single infectious disease [1]. In 2019, approximately 10 million people were diagnosed with TB and 1.4 million people died worldwide [1]. To prevent the spread of pulmonary TB, timely and effective anti-TB treatment is very important [2]. First-line anti-TB drugs include pyrazinamide (PZA), ethambutol (EMB), isoniazid (INH), and rifampin (RIF) [3-6]. When treating patients with TB, drug-induced liver injury (DILI) is the most frequent and serious side effect [7-10]. Among various populations, the incidence of TB-DILI ranges from 2.3% to 27.7% during anti-TB therapy [11-14]. Researchers have suggested that anti-TB drugs are hepatotoxic [11,15-18].

TB-DILI may result from direct toxic injury to hepatocytes by anti-TB drugs or their metabolites or immune-mediated liver injury and induction of hepatocyte apoptosis caused by anti-TB drugs that trigger multiple inflammatory immune pathways [11,19]. TB-DILI is characterized by a transient mild elevation of transaminases or acute hepatitis [20]. Fulminant hepatic failure is likely to develop in severe cases, whereas chronic hepatitis occurs in a minority of patients.

Currently, clinical liver tests usually include biochemical parameters of blood, such as transaminases including alanine transaminase (ALT), alkaline phosphatase, bilirubin, lactate dehydrogenase, and albumin, along with liver imaging and histopathologic evaluation. It is difficult to distinguish DILI from non-DILI on the basis of these indicators, since test results are largely consistent in DILI and non-DILI detection. In addition, clinical markers commonly used at present, accounting for neither differences in type and mechanisms of action of hepatotoxic drugs nor individual patients’ characteristics, only facilitate evaluation based on toxicity outcomes [21]. Therefore, identification of predictors at clinical stages and risk predictors of TB-DILI among patients has become an urgent and necessary task.

Previous studies have shown that TB-DILI is associated with some demographic characteristics and underlying chronic disease [12,22-26]. Patterson et al [27] suggested that an increase in pretreatment ALT and the gradient of ALT changes increase the risk of late TB-DILI. Thus, in addition to the cumulative anti-TB drug dose, ALT levels and demographic variable such as age, gender, education level, income, and BMI were included in our model as predictors. Various models are used to identify drugs associated with the risk of DILI at the preclinical stage [28]. Machine learning models have demonstrated strong predictive power and retained a simple form for communication with researchers [29-39]. XGBoost is a boosting ensemble machine learning algorithm that integrates a few classification and regression trees models to form a strong classifier [40,41]. It performs well in dealing with nonlinear and complex relationships among variables [42]. We designed an interpretable prediction model by using the XGBoost algorithm and identified the most robust and meaningful predictors of the risk of TB-DILI. Then, using these discriminative predictors, the machine learning model built an interpretable decision tree to provide early warning signals before TB-DILI occurs, so as to help clinicians adjust the medication plan in time and potentially reduce the possibility of TB-DILI. In this study, we retrospectively assessed 757 patients with TB who were registered for treatment in Nanshan District (Shenzhen, China) from 2014 to 2019.

## Methods

### Data

We extracted data on 757 pulmonary TB cases registered in the Hospital Information System of Shenzhen Nanshan Center for Chronic Disease Control from 2014 to 2019, including those that are smear-positive and undergoing initial treatment. Some patients did not have continuous treatment or were initially discharged from hospital and subsequently rehospitalized, resulting in the recorded treatment duration exceeding the normal range and unclear cumulative dosage of anti-TB drugs. Such abnormal cases are not able to contribute to predictions among patients receiving regular treatment. Thus, we selected 300 days as a time-window empirically on the basis of the typical course of TB treatment [1]. We excluded cases of TB-DILI that were recorded 300 days after the start of the anti-TB treatments. In total, data from 743 patients were finally included in the model. We defined patients as positive DILI cases in accordance with the American Thoracic Society criteria [11]: in the presence of hepatitis symptoms, the increase in ALT levels was 3-fold the normal upper limit, and in the absence of hepatitis symptoms, this increase was 5-fold the normal upper limit.

Patients’ demographic and clinical data included gender, age, weight, education level, income, height, hepatitis B status, diabetes status, cumulative anti-TB drug dose, and ALT levels. For patients who did not develop TB-DILI, we collected their total amount of prescribed anti-TB medication as of the latest hepatic examination. For patients who developed TB-DILI, we recorded their cumulative dose of anti-TB medication as of the time when TB-DILI was detected. In addition, we measured the patient’s most recent ALT levels before the last hepatic examination, and the average rate of change of the last 2 ALT levels tested before the final liver function test. We calculated the cumulative dose of each drug separately (PZA, RFP, EMB, and INH) for combination drug therapy.

Upon initiation of therapy, the patients were segregated to form the training and validation data sets. The data of patients admitted before April 2019 (607 patients and 186 smear-positive cases) and after April 2019 (136 patients and 95 smear-positive cases) were included in the training and validation data sets, respectively.

### Descriptive Statistics

Descriptive statistics were calculated for positive and TB-DILI cases. Demographic and laboratory data of the 2 groups were compared using 2-sample *t* tests for normally distributed continuous variables, the Kruskal–Wallis rank sum test for nonnormally distributed continuous variables, and chi-square tests for categorical variables. Missing values were omitted when tested for differences. Multimedia Appendix 1 shows the proportion of missing values for each variable.

### Prediction Model

We used the XGBoost algorithm for the prediction model [41]. XGBoost is a high-performance machine learning algorithm based on the tree boosting system [43-47]. It uses a sparsity-aware learning algorithm to process sparse data and weighted quantile sketch to approximate tree learning [41]. Since the decision tree is a simple classifier composed of hierarchically organized dichotomous determinations, its structure also demonstrates good interpretability [48-50]. In addition, the model can deal with missing values well. When the model searches for the best candidate split criteria for tree growth, they will also assign a default direction for the missing values on those nodes [41]. The interpretable criteria and high tolerance for missing data in the decision tree make the model robust and meaningful when dealing with clinical data. To obtain a model that can be conveniently applied in a clinical setting, we attempted to reduce the complexity of the model as much as possible. Hence, we choose the single-tree XGBoost algorithm as the prediction model.

To build the model, we first included all demographic and clinical data as predictors. The dependent variable is DILI status, which is a binary outcome. We trained the single-tree XGBoost algorithm with the training set. By considering each feature’s contribution for each tree in the model, we determined their relative importance to the tree model [51]. We repeated stratified 10-fold cross-validation 100 times to model on the training data set to obtain the mean value of each feature’s relative importance. Then, we arranged the top 10 predictors in accordance with their relative importance. The predictors were added into the model individually in descending order of relative importance to form 10 candidate models. We repeated stratified 10-fold cross-validation 100 times to the candidate models on the basis of the training data set to obtain the mean area under the receiver operating characteristic curve (AUC) and selected the model with the maximum AUC as the final model. Then, we trained the selected model with the whole training data set to obtain the interpretable decision tree. The detailed process of the stratified *k*-fold cross-validation and the parameters set in model is provided in Multimedia Appendix 1.

### Evaluation of Model Performance

We trained the model with the whole training data set and applied the model on the validation data set. We then evaluated the prediction results on the basis of the confusion matrix, which is a specific table to visualize the performance of a classification model [52]. In accordance with the confusion matrix, we calculated the value of the following evaluation indicators: precision, recall, F1 value, classification accuracy, and balanced error rate. Detailed descriptions of the formulae for the indicators are provided in Multimedia Appendix 1. To determine whether the model can send early an warning signal in time, we also calculated the duration from the timepoint when model sent the warning signal to the actual date of TB-DILI diagnosis among incorrectly classified cases. Meanwhile, we compared the performance (AUC) of the single-tree XGBoost algorithm with that of the multitree XGBoost algorithm, logistic regression, single-tree random forest algorithm, and multitree random forest algorithm through 10-fold cross-validation using the whole data set. We determined 95% CI values for AUC values with the DeLong method [53]. We applied selected variables to train the single-tree XGBoost model since variable selection is part of the whole algorithm. The complete data set was applied to train the other models. In addition, we applied multiple imputation by chained equations [54] to address missing data for logistic regression.

### Sensitivity Analysis

We selected 250 days and 350 days as alternative time windows to filter data. Then, we trained the model and compared the selected predictors. Performance (AUC) of the original model and that of 2 alternative models were also compared on the basis of the whole data set through 10-fold cross-validation. All analyses were performed with R (version 4.0.4, The R Foundation). The codes used in this study can be found in the GitHub repository [55].

## Results

**Figure 1:**
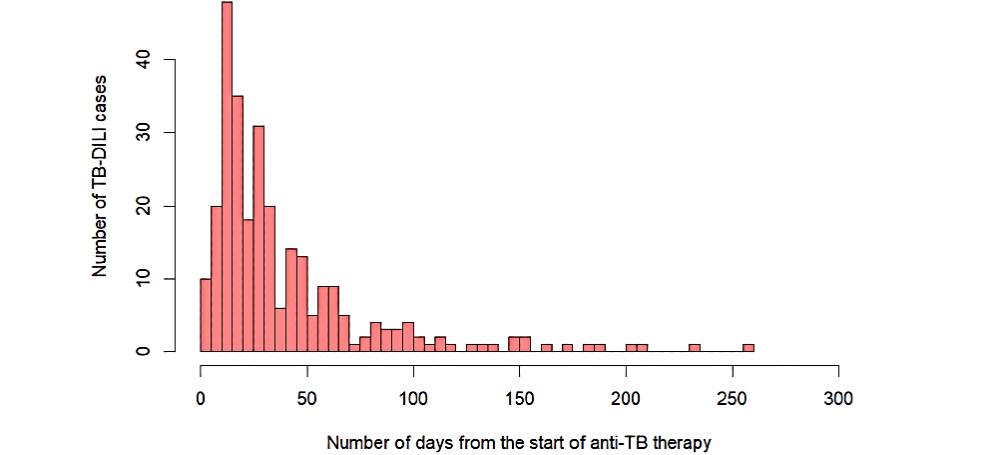
Days from tuberculosis treatment to the onset of drug-induced liver injury among the patients in our study. DILI: drug-induced liver injury, TB: tuberculosis.

In total, 743 patients were included in the analysis, of whom 281 (37.8%) and 462 (62.2%) were classified as TB-DILI–positive and –negative, respectively. Table 1 shows the descriptive statistics. The median age of patients was 30 (IQR 25-45) years, and 484 (65.1%) patients were male. Most patients (n=272, 43.5%) had a bachelor’s degree or higher education level. Median weight of the patients was 56 (IQR 50-63) kg and their median height was 168 (IQR 160-173) cm. In total, 24 (3.2%) patients had hepatitis B, and 69 (9.3%) patients had diabetes. The proportion of male patients who had DILI (n=281, 74.0%) was significantly higher than that of patients who did not have DILI (n=276, 59.7%). The most recently determined ALT level and average rate of change of the last 2 ALT measures of patients with DILI (27.0 U/L, IQR 17.0-34.0 U/L and 0.27 U/[Lday], IQR 0.0-0.6 U/[Lday], respectively) were significantly higher than those of patients who did not have DILI (11.0 U/L, IQR 8.3-16.0 U/L and 0.0 U/[Lday], IQR –0.1 to 0.1 U/[Lday], respectively). Figure 1 shows the number of TB-DILI cases on each day after the initiation of anti-TB treatment. The median time from treatment to the onset of DILI is 27 (IQR 15-48) days.

**Table 1:** Demographic and clinical characteristics of patients (N=743).

Characteristics			Overall		Negative cases (n=462)		Positive cases (n=281)		*P* value	
Males, n (%)			484 (65.1)		276 (59.7)		208 (74.0)		<.001	
Age^a^ (years), median (IQR)			30 (25-45)		30 (25-45)		31 (25-44)		.62	
Weight^a^ (kg), median (IQR)			56.0 (50.0-63.0)		55.00 (49.0-63.0)		57.0 (51.5-63.0)		.06	
**Education level, n (%)**										.55
	Lower than middle school	201 (32.2)		123 (33.1)		78 (30.8)				
	Middle school	152 (24.3)		93 (25.0)		59 (23.3)				
	Bachelor’s degree or higher	272 (43.5)		156 (41.9)		116 (45.8)				
Income^a^ (RMB^b^), median (IQR)			500,000 (300,000-800,000)		500,000 (300,000-800,000)		600,000 (500,000-1,000,000)		.002	
Height^a^ (cm), median (IQR)			168.0 (160.0-173.0)		167.0 (160.0-172.0)		168.0 (162.0-173.0)		.03	
Hepatitis B, n (%)			24 (3.2)		16 (3.5)		8 (2.8)		.81	
Diabetes, n (%)			69 (9.3)		48 (10.4)		21 (7.5)		.23	
BMI^a^, median (IQR)			20.0 (18.5-22.1)		19.9 (18.4-22.0)		20.2 (18.7-22.2)		.39	
Pyrazinamide dose^a^ (g), median (IQR)			16.8 (3.0-60.0)		24.0 (3.1-87.9)		5.4 (3.0-25.6)		<.001	
Rifampicin dose^a^ (g), median (IQR)			13.5 (1.3-67.5)		40.5 (5.5-94.5)		3.2 (1.2-12.6)		<.001	
Ethambutol dose^a^ (g), median (IQR)			18.7 (2.2-91.1)		50.3 (4.4-139.2)		5.3 (2.2-18.8)		<.001	
Isoniazid dose^a^ (g), median (IQR)			8.1 (0.8-37.0)		22.8 (3.6-58.3)		2.0 (0.6-6.5)		<.001	
Recent alanine transaminase measurement^a,c^ (U/L), median (IQR)			13.0 (10.0-23.0)		11.0 (8.3-16.0)		27.0 (17.0-34.0)		<.001	
Rate of change in alanine transaminase levels^a,d^ (U/[Lday]), median (IQR)			0.0 (–0.1 to 0.1)		0.00 (–0.1 to 0.1)		0.27 (0.0 to 0.6)		<.001	

^a^Nonnormally distributed variables.

^b^1 RMB=US $0.15.

^c^Patients’ most recently determined alanine transaminase level before the latest hepatic examination.

^d^Average rate of change of the patients’ last 2 alanine transaminase measures before the final liver function test (increment divided by the duration).

**Figure 2:**
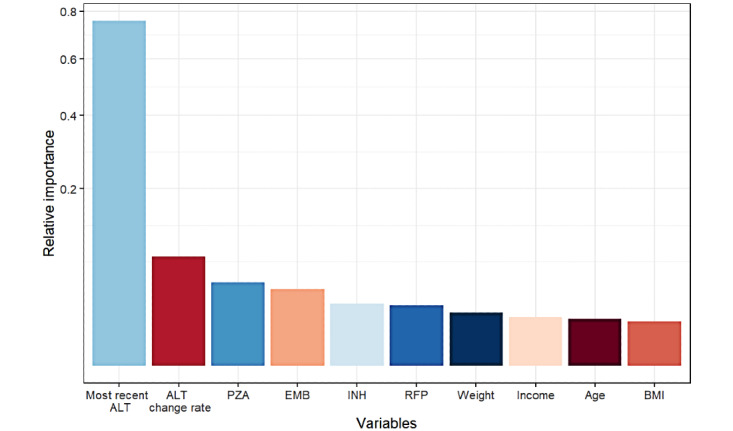
Top 10 important variables selected by the single-tree XGBoost model. ALT: alanine transaminase, EMB: ethambutol, INH: isoniazid, PZA: pyrazinamide, RFP: rifampicin.

**Figure 3:**
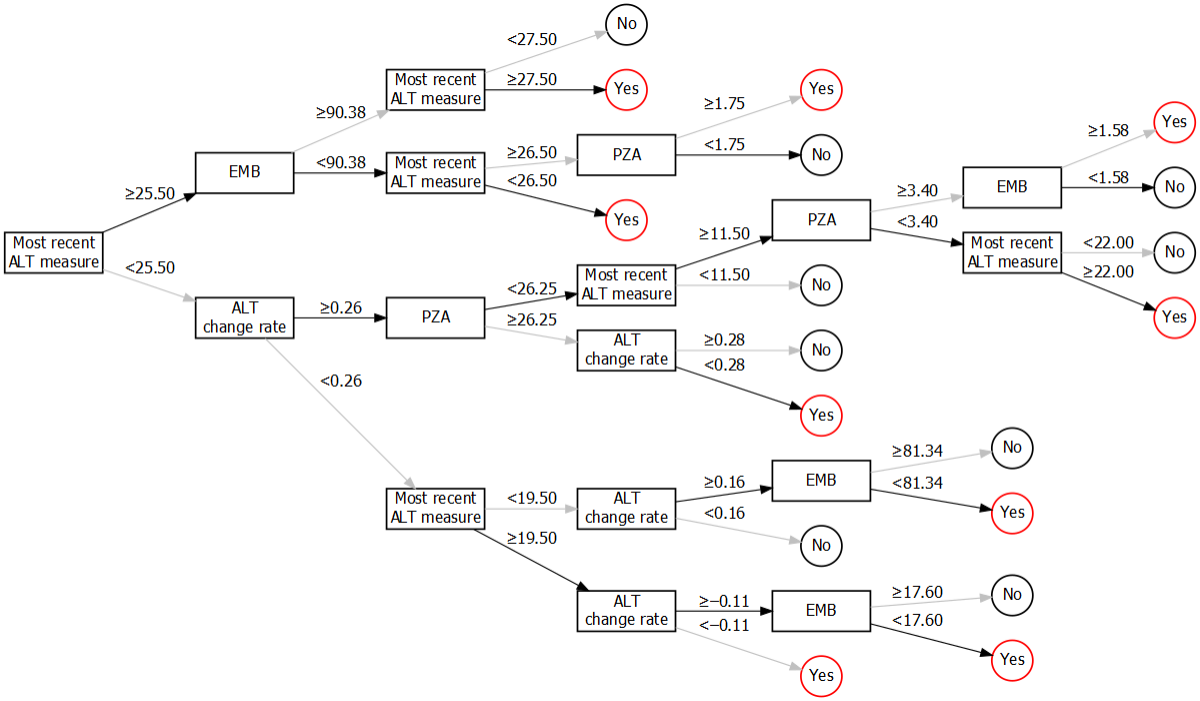
Detailed overview of the single decision tree of the model. The decision process starts from the left (most recent ALT measure) and ends at the right ("Yes": high risk of drug-induced liver injury or "No": low risk of drug-induced liver injury). Dichotomous determinations are made at every node in the decision tree. Cumulative doses of PZA and EMB are referenced. Black paths are the default direction for missing values. ALT: alanine transaminase, EMB: ethambutol, PZA: pyrazinamide, RFP: rifampicin.

Figure 2 shows the top 10 important variables selected by the single-tree XGBoost model. The most recent ALT levels were found to be the most important factor in the prediction process. We added 10 variables in the model individually to form 10 candidate models. After 10-fold cross-validation 100 times with the training and testing data sets, the model with 4 variables had the maximum AUC value (Table 2). Thus, we selected the model with 4 variables (the most recent ALT measure, average rate of change of the last 2 ALT measures, cumulative dose of PZA, and cumulative dose of EMB) as the final model. Then, we trained the selected model with the whole training data set. Figure 3 shows the content of a single decision tree of the model. The decision process starts from the most recent ALT test value, and then dichotomous determinations are made at each node in the decision tree; this process ends with outputting predictions (high or low risk of DILI).

**Table 2:** Summary of AUC^a^ values for candidate model.

Candidate model	Variables, n	AUC, mean (SD)
1	1	0.908 (0.043)
2	2	0.912 (0.040)
3	3	0.913 (0.041)
4 (selected model)	4	0.918 (0.040)
5	5	0.917 (0.040)
6	6	0.915 (0.040)
7	7	0.913 (0.040)
8	8	0.913 (0.041)
9	9	0.912 (0.041)
10	10	0.911 (0.041)

^a^AUC: area under the receiver operating characteristic curve.

**Figure 4:**
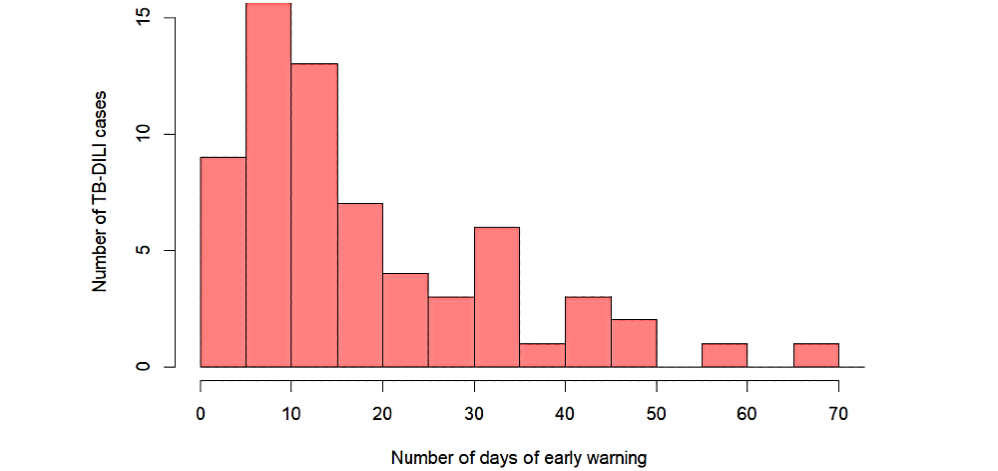
Number of days between the onset of drug-induced liver injury and the model providing warnings of high risk for patients with TB-DILI. TB-DILI: tuberculosis with drug-induced liver injury.

Table 3 summarizes the performance of the model on basis of the validation data set (136 cases). A total of 70 cases of DILI were correctly predicted, and 33 negative cases were successfully predicted. The model had a precision of 90%, recall of 74%, classification accuracy of 76%, balanced error rate of 77%, and F1 value of 81%. For correctly predicted cases, the median number of days between DILI onset and the provision of warnings of high risk by the model for the patients was 15 (IQR 7.3-27.5) days (Figure 4). Multimedia Appendix 1 shows a comparison of the performance of the single-tree XGBoost model and the multitree XGBoost model, logistic regression model, and multi- or single-tree random forest model on the whole data set, based on the receiver operating characteristic curve and the AUC. The multitree XGBoost model performed the best (AUC=0.940, 95% CI 0.924-0.956). The single-tree XGBoost model had an AUC of 0.912 (95% CI 0.890-0.935), which was very similar to that of the multitree model and higher than that of the rest of the models.

Table 4 shows the AUC values for candidate models under different time windows upon sensitivity analysis. Both final models under different time windows included the 4 most important predictors, same as those of our original model. The most recent ALT measure, average rate of change of patients’ last 2 ALT measures, and cumulative dose of PZA were identified as the best predictors in all 3 models. Nevertheless, our original model also selected the cumulative dose of EMB as an important predictor and, while being trained on the basis of 250-day and 350-day time windows, selected cumulative doses of RFP and INH, respectively. The performance of the 3 models is summarized in Multimedia Appendix 1, which shows that all 3 models have similar patterns of the receiver operating characteristic curve and provided largely consistent AUC values.

**Table 3:** Model performance^a^ with the validation data set.

Prediction or reference model	Yes	No
Yes, n	70	8
No, n	25	33

^a^Precision=90%, recall=74%, F1 value=81%, classification accuracy=76%, and balanced error rate=77%.

**Table 4:** Summary of AUC^a^ values for candidate models upon sensitivity analysis.

Candidate model	Variables, n	AUC of the model with a 250-day time window, mean (SD)	AUC of the model with a 350-day time window, mean (SD)
1	1	0.910 (0.040)	0.913 (0.042)
2	2	0.916 (0.039)	0.911 (0.040)
3	3	0.920 (0.039)	0.915 (0.041)
4 (selected model)	4	0.922 (0.039)	0.915 (0.041)
5	5	0.921 (0.039)	0.915 (0.041)
6	6	0.918 (0.038)	0.915 (0.042)
7	7	0.918 (0.039)	0.915 (0.041)
8	8	0.917 (0.039)	0.913 (0.041)
9	9	0.916 (0.039)	0.913 (0.041)
10	10	0.916 (0.039)	0.912 (0.041)

^a^AUC: area under the receiver operating characteristic curve.

## Discussion

### Principal Findings

Anti-TB drugs are one of the most common and effective means of treating TB in the clinical setting and can effectively control disease progression among patients with TB. Nevertheless, studies have suggested that patients are likely to develop DILI during the treatment process owing to the hepatotoxicity of anti-TB drugs [11,15-18] and long duration of TB treatment [56]. Clinicians often have difficulties in predicting the efficacy of anti-TB treatment as well as liver injury status in patients with TB. To identify reliable and accurate predictors and better predict DILI during TB treatment, we built the single-tree XGBoost machine learning model and selected variables with significant effects. Our model can provide suggestions to clinicians to adjust their medication regimens in a timely manner to avoid causing more severe liver injury. To our knowledge, this is the first time that XGBoost model has been applied to predict DILI at the clinical treatment stage.

Interestingly, the proportion of TB-DILI cases is significantly higher among men compared than among women (Table 1). This result is consistent with that of Chang et al [23], which suggested that males were 2.1-fold more likely to develop hepatotoxicity than females after being adjusted for age. We found that patients with DILI had significantly higher values for the most recent ALT measure and higher mean rates of change between the 2 most recent ALT measures than those without DILI (Table 1). Singanayagam et al [57] also demonstrated the association between pretreatment ALT and 2-week on-treatment ALT levels in patients with DILI.

Based on the results of variable selection, the significant predictors for predicting DILI are the most recent ALT measure, average change rate of the last 2 ALT measures, and the cumulative doses of PZA and EMB. According to the decision tree (Figure 3), the decision process of our model was to initially focus on the most recent ALT measure of a patient and then comprehensively evaluate the rate of change in ALT levels and the cumulative intake dose of both PZA and EMB to make predictions. A previous study [58] reported that the initial concentration of PZA and its metabolites are associated with hepatotoxicity [58]. Cao et al [59] suggested that combination therapy with PZA, INH, and RIF is likely to increase the risk of hepatotoxicity compared to monotherapy with INH and RIF. In addition, the addition of a low dose of PZA to a regimen of INH, RIF, and EMB did not significantly increase the incidence of DILI in the first 2 months of anti-TB therapy [60]. In the branches of our single-tree model (Figure 3), thresholds to determine whether the cumulative dose of anti-TB drugs contributed to the development of DILI under different situations were also provided.

Currently, various machine learning algorithms have been assessed for early detection of DILI and have shown to have a high prediction accuracy [61,62]. Xu et al [63] proposed a deep learning model, which achieved a classification accuracy of 86.9% in external validation for DILI prediction after training with a set of 475 samples. Dominic et al [64] combined mechanistic detection of hepatic safety with a Bayesian machine learning algorithm to build the model, which has a balanced accuracy of 86%, sensitivity of 87%, and specificity of 85%, thus improving the prediction of DILI risk. In addition, the XGBoost model was applied to increase the specificity of mass TB screening [65]. Our model also demonstrated the high prediction accuracy and interpretability of the XGBoost model at the clinical treatment stage.

Compared with alternative models, the multitree XGBoost model performed the best, as revealed from the AUC value upon cross-validation (Multimedia Appendix 1). The single-tree XGBoost model displayed similar performance to that of the multitree XGBoost model. Since the single-tree model is easier to interpret, takes up fewer computing resources, and provides predictions in a shorter period of time, the single-tree XGBoost model is more suitable in the clinical setting. In addition, the single-tree XGBoost model performed better than multitree random forest model and single-tree random forest model. The logistic regression model is also interpretable. Nevertheless, since linear models cannot directly process missing values, the missing clinical data could affect the performance of logistic regression. For multiple imputation, additional assumptions and prior information are required, which is likely to complicate the process and reduce the robustness of the model. In addition, sensitivity analysis has shown that our model has a consistently high prediction accuracy when trained with different time windows. Therefore, the single-tree XGBoost model is the most appropriate among all candidate models.

### Limitations

Our model also has some limitations of note. First, although the model identified the most meaningful predictors for the risk of TB-DILI, pathological conclusions should be made cautiously since the model was entirely driven by the data input. The model needs to be adjusted accordingly when the data are updated. Second, there is a lack of validation for our model on other data sets. Future studies could further explore these issues by applying the model in a combined larger data set. Inputting more data is likely to contribute to the identification of more effective predictors and generate higher prediction accuracy. In addition, it is also necessary to validate the model’s performance on an imbalanced data set to determine whether a further reweighting or resampling is needed to improve prediction accuracy.

### Conclusions

We developed a single-tree XGBoost model, which demonstrated the patients’ most recent ALT measure, average rate of change of patients’ 2 latest ALT measures, and cumulative doses of PZA and EMB as the best predictors for assessing the DILI risk. In the validation data set, the model displayed high accuracy (precision=90%, recall=74%, classification accuracy=76%, and balanced error rate=77%) and interpretability in predicting the TB-DILI cases. In addition, the median number of days between the model providing warnings of high risk among patients and DILI onset is 15 (IQR 7.3-27.5) days, which suggests that it is possible for clinicians to adjust the medication regimen by referring to the model’s prediction and avoid causing more serious liver injury.

## Appendix

Multimedia Appendix 1 Supplementary material.

## Abbreviations

ALT: alanine transaminase
AUC: area under the receiver operating characteristic curve
DILI: drug-induced liver injury
EMB: ethambutol
INH: isoniazid
PZA: pyrazinamide
RIF: rifampicin
TB: tuberculosis
