# Correction: Predicting Antituberculosis Drug–Induced Liver Injury Using an Interpretable Machine Learning Method: Model Development and Validation Study

**DOI:** 10.2196/32415

**Published:** 2021-08-13

**Authors:** Tao Zhong, Zian Zhuang, Xiaoli Dong, Ka Hing Wong, Wing Tak Wong, Jian Wang, Daihai He, Shengyuan Liu

**Affiliations:** 1 Department of Tuberculosis Control Shenzhen Nanshan Center for Chronic Disease Control Shenzhen China; 2 Department of Applied Mathematics The Hong Kong Polytechnic University Hong Kong China; 3 Department of Biostatistics University of California Los Angeles, CA United States; 4 Hong Kong Polytechnic University Shenzhen Research Institute Shenzhen China; 5 Research Institute for Future Food The Hong Kong Polytechnic University Hong Kong China; 6 Department of Applied Biology and Chemical Technology The Hong Kong Polytechnic University Hong Kong China

In “Predicting Antituberculosis Drug–Induced Liver Injury Using an Interpretable Machine Learning Method: Model Development and Validation Study” (JMIR Med Inform 2021;9(7):e29226) two corrections were made.

1. In the originally published article, author *Daihai He* was listed as the corresponding author. The corresponding author has been changed to *Shengyuan Liu* and the corrected details are as follows:

**Corresponding Author:**

Shengyuan Liu, PhD

Department of Tuberculosis Control

Shenzhen Nanshan Center for Chronic Disease Control

Nanshan District

Hua Ming Road No 7

Shenzhen, 518000

China

Phone: 86 13543301395

Email: jfk@sznsmby.com

2. For authors *Xiaoli Dong, Ka Hing Wong*, and *Wing Tak Wong*, the affiliation was originally listed as follows:

Xiaoli Dong^2^, PhD; Ka Hing Wong^2^, PhD; Wing Tak Wong^2^

Department of Applied Mathematics, Hong Kong Polytechnic University, Hong Kong, China

This has been corrected to:

Xiaoli Dong^5,6^, PhD; Ka Hing Wong^5,6^, PhD; Wing Tak Wong^5,6^

^5^Research Institute for Future Food, The Hong Kong Polytechnic University, Hong Kong, China

^6^Department of Applied Biology and Chemical Technology, The Hong Kong Polytechnic University, Hong Kong, China

The complete author information and affiliations in the corrected article are listed below.

Tao Zhong^1^*, BSc; Zian Zhuang^2,3,4^*, BSc; Xiaoli Dong^5,6^, PhD; Ka Hing Wong^5,6^, PhD; Wing Tak Wong^5,6^, PhD; Jian Wang^1^, BSc; Daihai He^2,4^, PhD; Shengyuan Liu^1^, PhD

^1^Department of Tuberculosis Control, Shenzhen Nanshan Center for Chronic Disease Control, Shenzhen, China

^2^Department of Applied Mathematics, Hong Kong Polytechnic University, Hong Kong, China

^3^Department of Biostatistics, University of California, Los Angeles, CA, United States

^4^Hong Kong Polytechnic University Shenzhen Research Institute, Shenzhen, China

^5^Research Institute for Future Food, The Hong Kong Polytechnic University, Hong Kong, China

^6^Department of Applied Biology and Chemical Technology, The Hong Kong Polytechnic University, Hong Kong, China

* these authors contributed equally

The correction will appear in the online version of the paper on the JMIR Publications website on August 13, 2021, together with the publication of this correction notice. Because this was made after submission to PubMed, PubMed Central, and other full-text repositories, the corrected article has also been resubmitted to those repositories.

