# Supplementary material for: Predicting Antituberculosis Drug–Induced Liver Injury Using an Interpretable Machine Learning Method: Model Development and Validation Study
Source: JMIR Med Inform. 2021 Jul 20;9(7):e29226. doi: 10.2196/29226 (PMC8335604; doi:10.2196/29226)
Supplement: Multimedia Appendix 1 [file medinform_v9i7e29226_app1.docx]

## Supplementary

### Stratified k-fold cross validation

In k-fold cross validation, the original dataset is stratified by positive/negative cases and then randomly divided into k subsamples of equal size. Among these subsamples, one subsample is treated as the validation dataset, and the rest k-1 sub samples are set as the training dataset for the model. This process is repeated K times so that each subsample can be set as the validation data once. Then we can obtain the estimate by calculating the mean or sum of K results from the fold. The advantage of k-fold cross validation is that each observation is only used for testing one time and results are not sensitive to the data partition method [1, 2].

### Confusion matrix

Confusion matrix is the contingency table which shows the relationship between the predicted results and the actual results. Both dimensions have the same level of results. Each column of confusion matrix represents the number of cases in that level of the actual class, while each row represents the number of cases in that level of the predicted class. In the field of machine learning, confusion matrix is usually used to evaluate the performance of classification algorithms [3].

### Performance evaluation indicator

TP: number of true positive cases in prediction

TN: number of true negative cases in prediction

FP: number of false positive cases in prediction

FN: number of false negative cases in prediction

Precision: $\frac{\mathrm{TP}}{(TP + FP)}$

Recall: $\frac{\mathrm{TP}}{(TP + FN)}$

F1: $\frac{2TP}{(2TP + FP + FN)}$

Classification accuracy: $\frac{(TP + TN)}{(TP + TN + FP + FN)}$

Balanced error rate: $\frac{1}{2}*(\frac{\mathrm{TP}}{\left( TP + FN \right)}+\frac{\mathrm{TN}}{\left( TN + FP \right)})$

| **Table S1.** Proportion of missing values for each variable | |
| --- | --- |
| **Variable name** | **Proportion of missing value (%)** |
| **EMB** | 0 |
| **PZA** | 0 |
| **RFP** | 0 |
| **INH** | 0 |
| **Gender** | 0 |
| **Age** | 0 |
| **Weight** | 15.75 |
| **Education level** | 15.88 |
| **Income** | 46.43 |
| **Height** | 17.09 |
| **Hepatitis B** | 0 |
| **Diabetes** | 0 |
| **BMI** | 16.96 |
| **ALT_updated** | 12.38 |
| **ALT_rate** | 30.01 |

| **Table S2.** Parameters used to train the XGBoost model | |
| --- | --- |
| **Parameter** | **Value** |
| Learning rate (eta) | 0.3 |
| max_depth | 6 |
| nround | 1 |
| eval_metric | logloss |


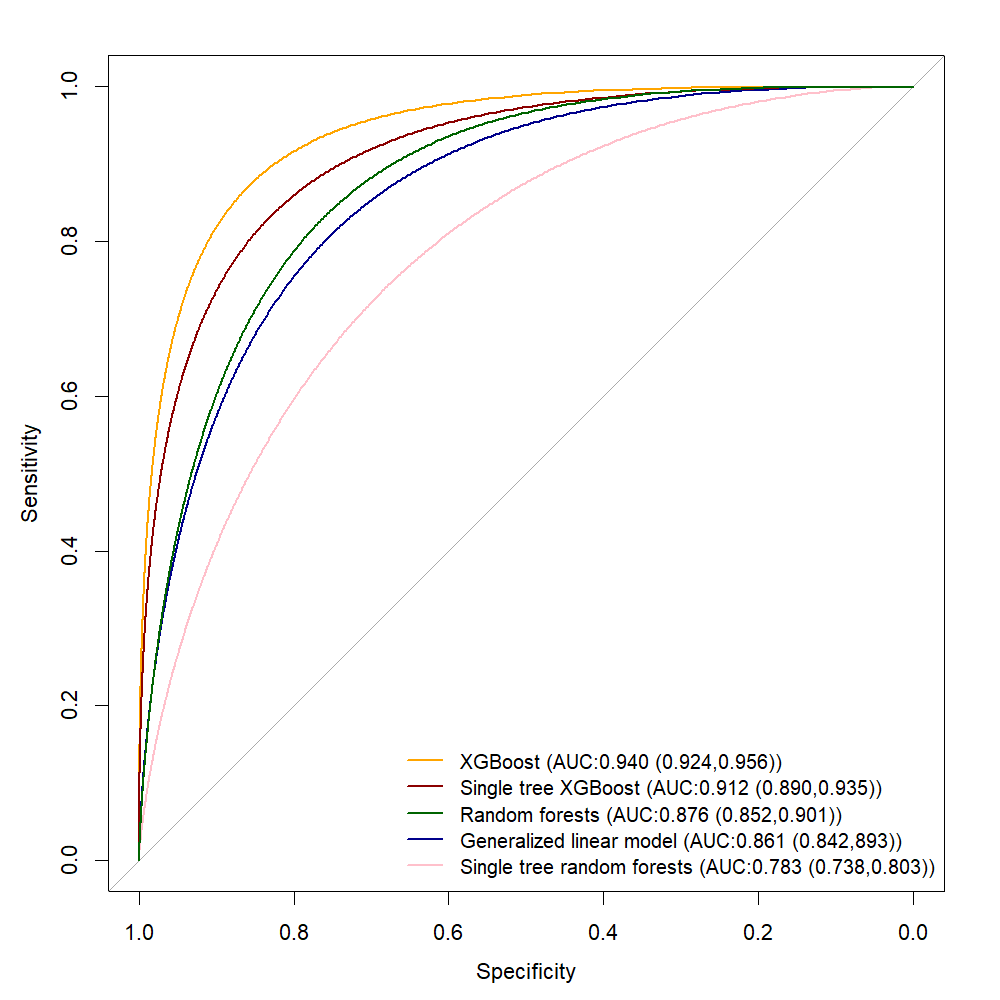


Figure S1. Receiver operating characteristic curve (ROC) and Area under the Curve of ROC (AUC) (95% CI) for different models.


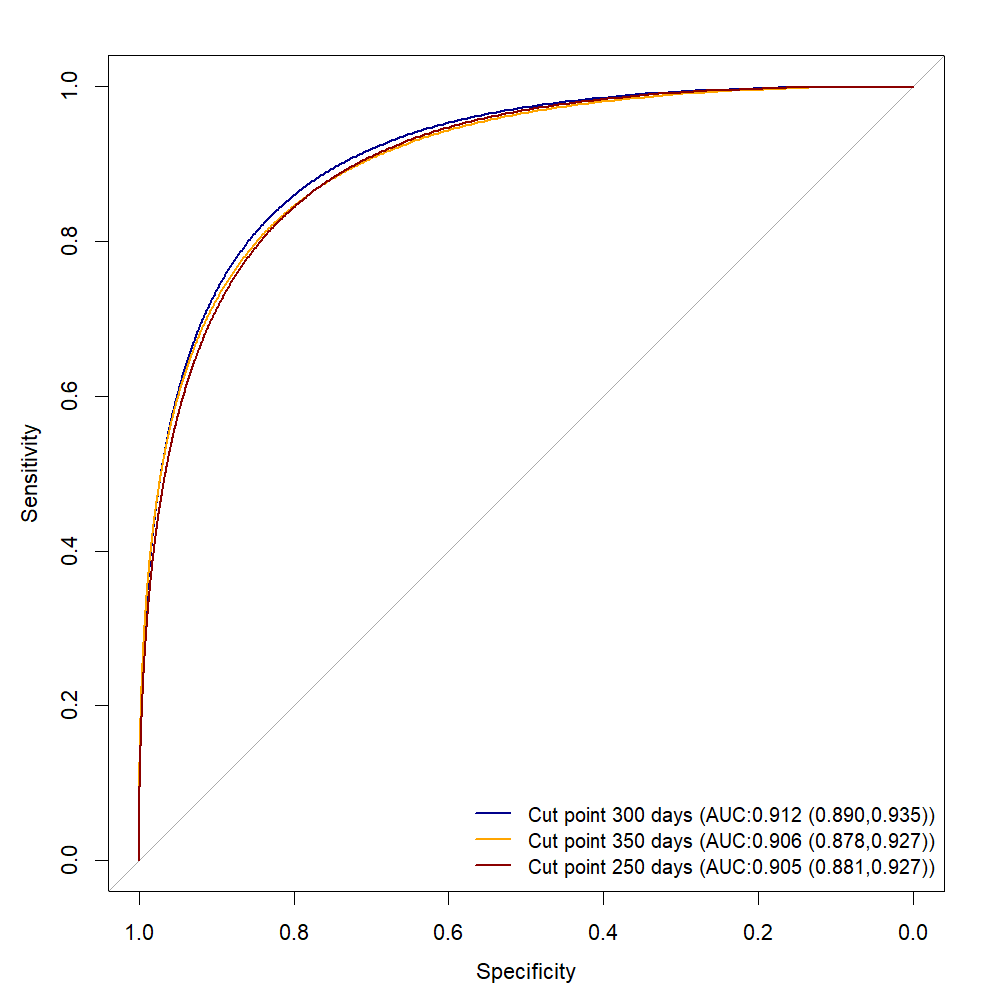


Figure S2. Receiver operating characteristic curve (ROC) and Area under the Curve of ROC (AUC) (95% CI) for original model and two alternative models in sensitivity analysis.

## Reference

1. Stone M. An Asymptotic Equivalence of Choice of Model by Cross-Validation and Akaike's Criterion. Journal of the Royal Statistical Society Series B (Methodological). 1977;39(1):44-7.

2. Stone M. Cross-Validatory Choice and Assessment of Statistical Predictions. Journal of the Royal Statistical Society: Series B (Methodological). 1974 1974/01/01;36(2):111-33. doi: https://doi.org/10.1111/j.2517-6161.1974.tb00994.x.

3. Powers DMW. Evaluation: from precision, recall and F-measure to ROC, informedness, markedness and correlation. arXiv preprint arXiv:201016061. 2020.
